# Clinical utility of circulating cell-free Epstein–Barr virus DNA in patients with gastric cancer

**DOI:** 10.18632/oncotarget.15675

**Published:** 2017-02-24

**Authors:** Katsutoshi Shoda, Daisuke Ichikawa, Yuji Fujita, Kiyoshi Masuda, Hidekazu Hiramoto, Junichi Hamada, Tomohiro Arita, Hirotaka Konishi, Toshiyuki Kosuga, Shuhei Komatsu, Atsushi Shiozaki, Kazuma Okamoto, Issei Imoto, Eigo Otsuji

**Affiliations:** ^1^ Division of Digestive Surgery, Department of Surgery, Kyoto Prefectural University of Medicine, Kamigyo-ku, Kyoto 602-8566, Japan; ^2^ Department of Human Genetics, Graduate School of Biomedical Sciences, Tokushima University, Tokushima 770-8503, Japan

**Keywords:** gastric cancer, Epstein–Barr virus, liquid biopsy

## Abstract

Recent comprehensive molecular subtyping of gastric cancer (GC) identified Epstein–Barr virus (EBV)-positive tumors as a subtype with distinct salient molecular and clinical features. In this study, we aimed to determine the potential utility of circulating cell-free EBV DNA as a biomarker for the detection and/or monitoring of therapeutic response in patients with EBV-associated gastric carcinoma (EBVaGC). The EBV genes-to-ribonuclease P RNA component H1 ratios (EBV ratios) in the GC tumors and plasma samples were determined by quantitative real-time polymerase chain reaction in 153 patients with GC, including 14 patients with EBVaGC diagnosed by the conventional method. Circulating cell-free EBV DNA was detected in 14 patients with GC: the sensitivity and specificity of detection were 71.4% (10/14) and 97.1% (135/139), respectively. Plasma EBV ratios were significantly correlated with the size of EBVaGC tumors, and the plasma EBV DNA detected before surgery in EBVaGC cases disappeared after surgery. Patients with EBVaGC may have a better prognosis, but circulating cell-free EBV DNA had no or little impact on prognosis. In addition, repeated assessment of the plasma EBV ratio in EBVaGC showed a decrease and increase in plasma EBV DNA after treatment and during tumor progression/recurrence, respectively. These results suggest the potential utility of circulating cell-free DNA to reveal EBV DNA for the identification of the EBVaGC subtype and/or for real-time monitoring of tumor progression as well as treatment response in patients with EBVaGC.

## INTRODUCTION

Gastric cancer (GC) is the fifth most commonly diagnosed cancer and the third leading cause of cancer-related mortality worldwide [1]. Recent comprehensive studies have characterized GC as a heterogeneous disease, and its molecular classification has become a major concern in GC research with respect to using it as a guide for developing curative targeted agents against GC. One type of GC is Epstein–Barr virus (EBV)-associated gastric carcinoma (EBVaGC), which has been classified as the molecular subtype of tumors positive for EBV in The Cancer Genome Atlas project [2] and enriched in the molecular subtype of microsatellite stable/TP53-active tumors by the Asian Cancer Research Group [3]. Because the high programmed death ligand 1/2 expression and immune pathway activation signature in EBVaGCs strongly justify targeting these molecules/pathways via immunotherapy, the diagnostic screening and monitoring of EBVaGC might prove fruitful in selecting candidates for immunotherapy [4].

To date, it has been shown that “liquid biopsy” using circulating cell-free DNA (cfDNA) could constitute a new paradigm for the study of clonal evolution in human cancers [5]. We previously demonstrated the potential utility of cfDNA as a resource for a repeatable and noninvasive evaluation of the human epidermal growth factor receptor 2 amplification status in GC [6, 7]. Although monitoring circulating cell-free EBV DNA can be an effective method for distinguishing disease-associated EBV reactivation from incidental presence of EBV in benign B lymphocytes as well as for diagnostic screening and monitoring of EBV-associated diseases such as Hodgkin's lymphoma, Burkitt's lymphoma, and nasopharyngeal carcinoma [8–10], little has been done to evaluate the role of EBV testing in the diagnosis and monitoring of GC [11]. In EBVaGC, all tumor cells harbor the clonal EBV genome [12], suggesting that the EBV genome is useful as a reliable and specific biomarker of cancer cells as common founder mutations. Indeed, the possibility of using circulating EBV DNA as a tumor marker for EBVaGC has been reported [13]. However, no studies have examined the dynamics of circulating EBV DNA during the clinical course in GC to determine its clinical significance in the diagnosis and monitoring of EBVaGC.

Therefore, in the present study, we attempted to quantitatively detect circulating cell-free EBV DNA in the plasma of GC patients (the primary endpoint) and examin its clinical potential as a tool for the diagnosis of EBVaGC and repeatable and non-invasive monitoring of tumor progression and therapeutic effects in patients with EBVaGC (the secondary endpoint).

## RESULTS

### Detection of EBV DNA in tumor tissues and plasma samples of GC patients using real-time quantitative polymerase chain reaction (rqPCR)

In the paraffin-embedded tissue sections, the established assay for EBV targets EBV-encoded small RNAs (EBER) by *in situ* hybridization (ISH). Among a total of 153 GC patients (Table 1), 14 were EBER positive (Supplementary Figure 1A). A previous study showed that EBV DNA levels generally reflect the EBER status, and a panel of at least two rqPCR assays is recommended for the sensitive identification of the infected cancers [14]. Therefore, latent membrane protein 1 (LMP1) and Epstein–Barr nuclear antigen 1 (EBNA1) levels were assessed to detect the presence of EBV DNA, and EBV DNA positivity was defined as a positive result in both the rqPCR assays. EBV DNA was detected in 21 GC tissue samples by rqPCR, including all 14 cases showing EBER positivity in ISH (sensitivity = 1.000, specificity = 0.677; Supplementary Figure 1B), whereas EBV DNA was not detected in all 153 non-tumorous gastric tissue samples of patients with GC. Detection for plasma EBV DNA was negative in all 50 healthy volunteers, whereas EBV DNA was positively identified in 14 patients with GC, ten of which had EBVaGC (sensitivity = 0.714, specificity = 0.971; Supplementary Figure 1B). Plasma LMP1 DNA was detected in 15 GC cases, including all 14 cases positive for plasma EBNA1 DNA (Supplementary Table 1). EBV DNA was not detected in the peripheral blood leukocytes of the 14 EBVaGC patients. In the ten patients positive for EBV DNA in both the GC tumor and plasma samples, the plasma EBV ratios were significantly correlated with the tissue EBV ratios (ρ = 0.7576, *p* = 0.011, Spearman's analysis, Supplementary Figure 2).

**Table 1:** Clinicopathological features of 153 gastric cancer (GC) patients with analysis of Epstein-Barr virus (EBV)

Variables	n	Tumor EBV DNA^a^					Plasma EBV DNA^b^				
		Negative (%)		Positive (%)		*P* value	Negative (%)		Positive (%)		*P* value
Total	153	132	(86.3)	21	(13.7)		139	(90.8)	14	(9.2)	
Sex											
Male	95	80	(84.2)	15	(15.8)	0.2424	84	(88.4)	11	(11.6)	0.1477
Female	58	52	(89.7)	6	(10.3)		55	(94.8)	3	(5.2)	
Age (years)											
<70	83	76	(91.6)	7	(8.4)	**0.0331**	79	(95.2)	4	(4.8)	**0.0405**
≥70	70	56	(80.0)	14	(20.0)		60	(85.7)	10	(14.3)	
Location											
Upper	57	47	(82.5)	10	(17.5)	0.2961	50	(87.7)	7	(12.3)	0.2259
Middle or lower	96	85	(88.5)	11	(11.5)		89	(92.7)	7	(7.3)	
Histopathological predominance^c^											
Differenciated	79	72	(91.1)	7	(8.9)	0.0576	75	(94.9)	4	(5.1)	0.0621
Undifferenciated	74	60	(81.1)	14	(18.9)		64	(86.5)	10	(13.5)	
Size (mm)											
<70	76	65	(85.5)	11	(14.5)	0.4870	72	(94.7)	4	(5.3)	0.0834
≥70	77	67	(87.0)	10	(13.0)		67	(87.0)	10	(13.0)	
Lymphatic invasion											
negative	37	30	(81.1)	7	(18.9)	0.2138	31	(83.8)	6	(16.2)	0.0875
positive	116	102	(87.9)	14	(12.1)		108	(93.1)	8	(6.9)	
Venous invasion											
negative	54	44	(81.5)	10	(18.5)	0.1524	46	(85.2)	8	(14.8)	0.0691
positive	99	88	(88.9)	11	(11.1)		93	(93.9)	6	(6.1)	
Depth of tumor invasion^d^											
pT1/2/3	83	69	(83.1)	14	(16.9)	0.1602	73	(88.0)	10	(12.0)	0.1415
pT4	70	63	(90.0)	7	(10.0)		66	(94.3)	4	(5.7)	
N stage^d^											
pN0	33	26	(78.8)	7	(21.2)	0.1315	27	(81.8)	6	(18.2)	0.0520
pN1-3	120	106	(88.3)	14	(11.7)		112	(93.3)	8	(6.7)	
pStage^d^											
pI/pII	56	48	(85.7)	8	(14.3)	0.5297	50	(89.3)	6	(10.7)	0.4058
pIII	97	84	(86.6)	13	(13.4)		89	(91.8)	8	(8.2)	
Tissue EBER^e^											
Negative	139	132	(95.0)	7	(5.0)	**<0.0001**	135	(97.1)	4	(2.9)	**<0.0001**
Positive	14	0	(0.0)	14	(100.0)		4	(28.6)	10	(71.4)	
Tumor EBV DNA^a^											
Negative	132	**-**		**-**			128	(97.0)	4	(3.0)	**<0.0001**
Positive	21	**-**		**-**			11	(52.4)	10	(47.6)	
Plasma EBV DNA^b^											
Negative	139	128	(92.1)	11	(7.9)	**<0.0001**	-		-		
Positive	14	4	(28.6)	10	(71.4)		-		-		

*P* values are from χ^2^ or Fisher's exact test and the results were considered statistically significant at P < 0.05. Statistically significant values are in boldface type.

^a^ Tumor EBV DNA was determined by rqPCR using *LMP1* and *EBNA1* primers.

^b^ Plasma EBV DNA was determined by real-time quantitative polymerase chain reaction (rqPCR) using latent membrane protein 1 (*LMP1*) and Epstein-Barr nuclear antigen 1 (*EBNA1*) primers.

^c^ Differentiated: tubular adenocarcinoma or papillary adenocarcinoma.

Undifferentiated: poorly differentiated adenocarcinoma, signet-ring carcinoma, or mucinous adenocarcinoma.

^d^ Disease stage was defined in accordance with the International Union Against Cancer 7th tumor-lymph node-metastases classification using surgical-pathologic findings.

^e^ Tissue EBV was determined by *in situ* hybridization as described in the text.

### Relationships between clinicopathological factors and plasma EBV ratios determined by rqPCR

**Figure 1:**
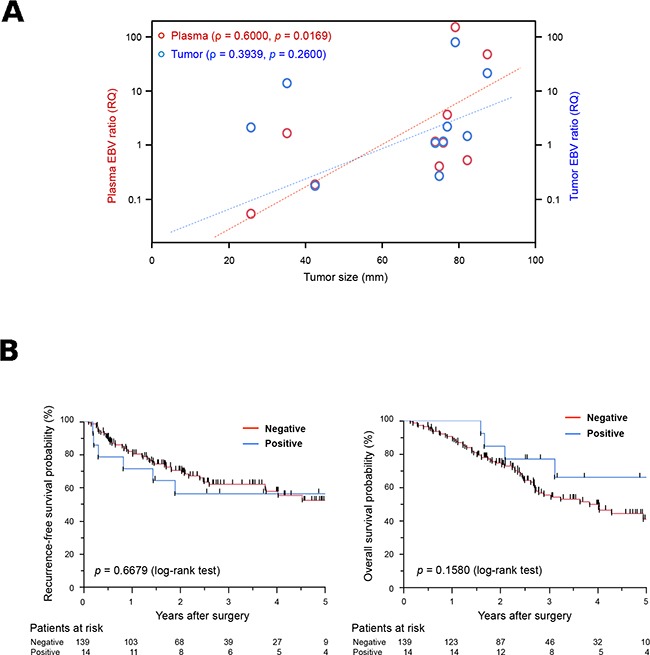
Potential of plasma Epstein-Barr virus (EBV) ratio for use in the detection of EBV DNA in cell-free DNA (cfDNA) samples **(A)** Relationship between tumor size and the EBV ratio in patients with gastric cancer (GC). Red and blue circles represent plasma and tumor EBV ratios for each patient, respectively. Plasma EBV ratios were significantly correlated with tumor sizes in patients with GC (ρ = 0.600, *p* = 0.0169, Spearman's correlation); however, tumor EBV ratios were not (ρ = 0.3939, *p* = 0.2600). RQ; relative quantitative value. **(B)** Kaplan-Meier curves for recurrence-free survival rates (left) and overall survival rates (right) of GC patients according to the presence of EBV DNA in the plasma. The log-rank test was used for statistical analysis. *P* < 0.05 was considered statistically significant.

The relationships between clinicopathological characteristics and plasma and tumor EBV DNA positivities in all 153 patients with GC are summarized in Table 1. A positive identification of EBV DNA in the plasma was associated with older patients; EBER-positive GC tumors were more frequently observed in older GC patients in our cohort (Supplementary Table 1). Positive identifications of plasma EBV DNA tended to be inversely correlated with venous invasion and N stage because EBER-positive GC tumors also showed a similar tendency (Supplementary Table 1). Notably, positive identifications of plasma EBV DNA tended to be positively correlated with tumor size; however, positive identifications of tissue EBV DNA and EBER did not show this tendency (Table 1, Supplementary Table 2). All four EBER-positive cases, in which plasma EBV DNA was not detected, were smaller than 70 mm (Supplementary Table 3). A similar tendency was observed between size and plasma EBV DNA status in tumor EBV DNA positive cases (Supplementary Table 3). In the ten cases with positive identification of both plasma EBV and tumor EBER, plasma EBV ratios were highly and significantly correlated with tumor sizes (ρ = 0.600 and *p* = 0.0169, Spearman's correlation; Figure 1A); however, tumor EBV ratios did not show this relationship (ρ = 0.3939, *p* = 0.2600), suggesting that the amount of cfDNA from EBV-positive tumors increases in a tumor size dependent manner. In the total of 153 cases, the median follow-up period after surgery was 31 months (range = 5–85 months). There was no significant difference in the overall survival (OS) and recurrence-free survival (RFS) between the plasma EBV-positive and plasma EBV-negative patients (Figure 1B), although OS rates were significantly higher in patients with EBVaGC in GC tissue (Supplementary Figure 3).

### Dynamics of the plasma EBV ratio during therapeutic course

**Figure 2:**
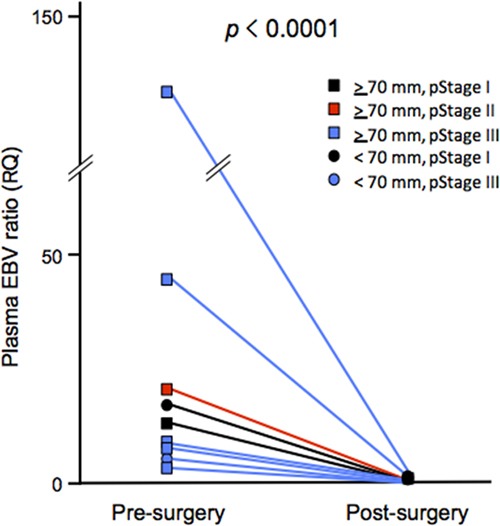
Dynamics of the plasma Epstein-Barr virus (EBV) ratio before and after surgery Sets of plasma cfDNA before and after surgery were available for nine patients with EBVaGC showing positive results of pre-operative plasma EBV DNA. Squares and circles represent tumors ≥70 mm and those <70 mm, respectively. Black, red, and blue colors indicate pathological stages (pStage) I, II, and III, respectively.

Plasma EBV ratios were comparable between pre- and paired post-operative samples in nine of 14 plasma EBV-positive cases. All nine cases showed positive results for both plasma EBV and tumor EBER. The ratios in the post-operative samples were also significantly lower than those in the pre-operative samples (n = 9; *p* < 0.0001, paired Wilcoxon *t*-test), and none of the post-operative plasma samples were positive for EBV DNA (Figure 2), thereby supporting the reproducibility of the rqPCR-based measurement for quantitative evaluation of cell-free EBV DNA. In addition, this result also supports that the plasma EBV ratio reflects the tumor burden of EBVaGC.

**Figure 3:**
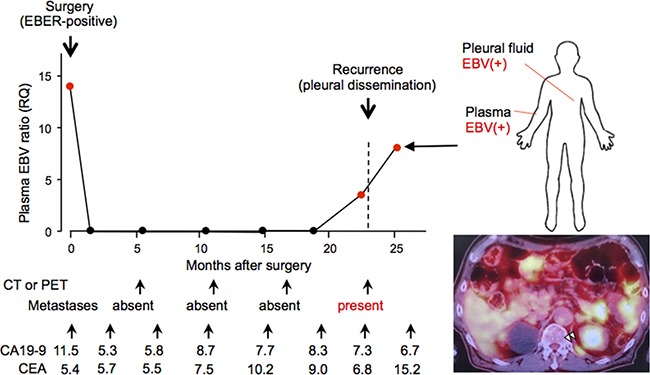
Longitudinal analysis of the clinical history of a case whose plasma Epstein-Barr virus (EBV) ratio was monitored repeatedly before and after surgery A 73-year-old male patient underwent surgery for gastric cancer of pathological stage T1, N0, M0, and stage IA. His plasma EBV ratio was high before surgery, and a decreased level was maintained until a month before pleura dissemination was diagnosed by positron emission tomography (right lower). EBV DNA was also detected in the pleural fluid at the time of recurrence (right upper). Note that neither carcinoembryonic antigen nor serum carbohydrate associated antigen values reflect the clinical history in this case.

We performed repeated measurements of the plasma EBV ratio before and after surgery in one patient with recurrence of EBVaGC, and compared those results with the patient's clinical history (Figure 3). In this case, the increase in the plasma EBV ratio was detected before operation, and the plasma EBV was not continuously detected after surgery. The increase in the plasma EBV ratio was detected a month before clinically apparent recurrence, and positivity for plasma EBV was continuously observed during the progression of metastatic tumors with little or no response to chemotherapy. The recurrent tumor was indicated by Positron Emission Tomography (PET) and cytopathological examination of the pleural fluid, with EBV DNA also detected in the pleural fluid. The levels of the conventional tumor marker CA19-9 were continuously lower than the cut-off values, and carcinoembryonic antigen (CEA) was not reflected in the dynamics of tumor progression during the entire clinical course. This result suggested that plasma EBV DNA is a sensitive marker for recurrence in patients with EBVaGC.

## DISCUSSION

The concept of detecting tumor-specific molecular alterations by analyses of bodily fluids, including peripheral blood, in cancer patients is termed “liquid biopsy” [15]. This new concept is expected to not only provide noninvasive diagnostic methods but also realize real-time monitoring of tumors by overcoming the issue of tumor heterogeneity in cancer care. In the present study, we utilized circulating cfDNAs in the plasma samples for the detection of EBV-related DNA because the major sources of cfDNA are considered to be the apoptotic and necrotic cells in tumors. The plasma EBV DNA was detected in 10 of 14 patients with EBVaGC but was not detected in 135 of 139 non-EBVaGC patients and all 50 healthy volunteers, as previously reported [13]. All four tumor EBER-positive cases, which showed negative plasma EBV DNA, were smaller than 70 mm in size, and significant correlation was observed between the tumor size and the plasma EBV ratio in plasma EBV DNA-positive patients with EBVaGC, which suggests that the amounts of cfDNA from GC cells in some of the smaller EBVaGC tumors were not sufficient to detect EBV DNA using rqPCR. However, the copy number of episomal EBV DNA in GC cells in each tumor is also the factor associated with the amount of cell-free EBV DNA from tumors. Therefore, the quantitative detection of cell-free EBV DNA in the plasma sample reflects tumor burden, and any method with higher sensitivity might allow more accurate detection of EBVaGC tumors. Four cases with negative EBER ISH and EBV DNA in GC tissues were positively identified for plasma EBV DNA. A previous study suggested the possibility that EBV could infect the noncarcinomatous gastric mucosa of chronic atrophic gastritis [13, 16]. A possibility exists that such nonspecific EBV infection had an influence on the discrepancy, although none of 153 cases of GC were positively identified for tumor EBV DNA in non-tumorous stomach tissues.

The EBV DNA was not detected in the postoperative plasma samples in all EBVaGC patients tested positive for preoperative plasma EBV DNA, indicating that our rqPCR-based detection of plasma EBV DNA is a highly specific method for monitoring response to treatment for EBVaGC. Although the stability of cfDNA is not well understood, circulating cfDNA appears to be rapidly cleared; the mean half-life of cell-free fetal DNA was previously estimated to be 16 min [17] and more recently to be 1 h in the initial rapid phase and 13 h in the subsequent slow phase [18]. A study regarding the kinetics and clearance of circulating EBV DNA may indicate equivalent mechanisms in the EBV-related tumor, such as nasopharyngeal carcinoma [19].

In comparison with the results of EBER analysis by ISH, the EBV DNA analysis of tissues showed a higher positivity rate. All the EBER-positive cases were positively identified for tissue EBV DNA, and an additional seven cases were found to only have EBV DNA in tumor samples. Although methods for targeting EBV DNA using two rqPCR assays are known to reflect the EBER status with a high sensitivity and specificity [14], the difference in sensitivity between the ISH and rqPCR assays might be one of the reasons responsible for this discrepancy, and rqPCR targeting episomal EBV DNA might detect latent EBV DNA in some cases of GC.

Our prognostic analysis demonstrated that there was no significant difference in the OS and RFS between plasma EBV-positive and -negative patients, whereas the presence of EBV in GC tissue had a favorable impact on the OS of GC patients, although effects on OS and RFS were opposite. Although EBVaGC has been reported to present a relatively favorable prognosis in the Asian population [20] and international analysis [21], the prognostic significance of EBVaGC remains controversial [22]. In a clinicopathological study in the Netherlands, cases of EBVaGC showed a better prognosis than negative cases [23]. In the present study, EBVaGC accompanied lymph node metastasis in a significantly lower frequency than EBV-negative GC (Supplementary Table 2), suggesting that cases of EBVaGC may have a better prognosis, although the numbers of cases were insufficient for prognostic analysis in previous studies as well as our cases. Although it remains unclear why circulating cell-free EBV DNA had no or little impact on prognosis in GC, it is possible that EBVaGC cases associated with better prognosis were included in plasma EBV-negative cases. Indeed, all four plasma EBV-negative EBVaGC cases had tumors smaller than 70 mm and relatively longer OS with no death in the observation period (range, 1.07–3.50 years; median, 1.46 years). Further study using a larger and independent cohort will be required to determine the prognostic impact of EBVaGC, as well as the plasma EBV DNA measurement.

The plasma EBV ratio decreased immediately after surgery, was elevated before an expression of markers found in various diagnostic imaging tools, and continuously increased with the progression of recurrent disease (Figure 3). These results indicate that the detection of plasma EBV in EBVaGC reflects tumor burden and could be a sensitive and repeatable marker for tumor progression and therapeutic effect for patients with EBVaGC at a low cost (reagent cost, <10 dollars per test), although its usefulness was proven only in one patient. Further studies using a larger number of EBVaGC patients and a prospective study design are required in the future. In addition, more sensitive methods without increasing the cost should be developed to monitor tumor burden repeatedly in EBVaGC patients as a clinical test.

## MATERIALS AND METHODS

### Patients and samples

The present study was performed in accordance with the Declaration of Helsinki protocols and was approved by the local Ethics Committee. All the subjects gave their written informed consent before participating. The samples were blinded for analyses and the patients were informed that the results would not be made available to them. The study cohort included 153 GC patients who underwent surgery, through which both tumor and plasma samples were collected between January 2008 and October 2014 at the Kyoto Prefectural University of Medicine Hospital (Table 1). Control plasma samples were obtained from 50 healthy adult volunteers by standard antecubital venous punctures. Patient demographic data and details regarding tumor recurrence and subsequent management were recorded. Computed tomography (CT) imaging was performed and reviewed in a blinded manner to document treatment responses according to the Response Evaluation Criteria in Solid Tumors (RECIST), version 1.1 [24]. The pathological classification of the tumors was performed according to the Union for International Cancer Control classification [25].

### Sample preparation and DNA isolation

A 7 mL ethylenediaminetetraacetic acid-blood sample was obtained from each patient before surgery and from 50 healthy volunteers. Samples were also collected from nine patients one month after surgery and from one patient during the post-operative period. The plasma was immediately separated from the cellular fraction using a three-spin protocol as described previously [26], following which the samples were stored at −80°C for further processing. Circulating cfDNA was isolated from 2 mL of the plasma sample using the QIAamp Circulating Nucleatic Acid Kit (Qiagen, Hilden, Germany). Genomic DNA from the cancerous gastric tissues was extracted from three slices (5 μm thick) of formalin-fixed, paraffin-embedded (FFPE) tissues using the QIAamp DNA FFPE Tissue Kit (Qiagen, Hilden, Germany) according to the manufacturer's protocols. We also extracted genomic DNA from peripheral blood leukocytes using the Gentra Puregene Blood Kit (Qiagen, Hilden, Germany).

### *In situ* hybridization (ISH) of EBV-encoded small RNAs (EBER)

Two adjacent sections were stained with hematoxylin and eosin by ISH for EBER with the inclusion of known EBER-positive and -negative tumors as external controls. Hybridization was performed using the Leica Bond system with 5 min of protease digestion and 2 h of probe hybridization. A tumor was interpreted as EBV negative if EBER staining was undetected or only localized to benign-appearing lymphoid cells, and as EBV positive if EBER staining was localized to the nucleus of malignant epithelial cells [14]. EBER-positive patients were determined to have EBVaGC.

### Quantitative analysis of EBV DNA using real-time quantitative polymerase chain reaction (rqPCR)

All rqPCR amplifications were performed in triplicate using the Applied Biosystems® Step one Real Time PCR system (Life Technologies, Carlsbad, CA, USA) according to the manufacturer's protocols. Using ribonuclease P RNA component H1 (*RPPH1*) as an internal control for the quantitative analysis of EBV DNA [6, 7], plasma EBNA1-to-*RPPH1* and plasma LMP1-to-*RPPH1* ratios were determined using the rqPCR assays. To determine EBV ratio, the ΔCt value (average Ct value of the target gene minus the average Ct value of the reference gene) was determined and used to calculate the ΔΔCt value for each DNA sample with a mean relative quantitation (RQ) value. RQ values were calculated from 2^−ΔΔCt^. EBV positivity was defined as the case wherein the Ct values of both target genes were detectable, that is, if the any Ct value was undetectable, the case was considered to be EBV negative. Only in cases positive for tissue or plasma EBV DNA, the tissue or plasma EBV ratio was calculated using the average of the EBNA1-to-*RPPH1* ratio and the plasma LMP1-to-*RPPH1* ratio.

The following primers were used for the rqPCR assays: LMP1 forward (5′-CAGTCAGGCA AGCCTATGA-3′), LMP1 reverse (5′-CTGGTTCCGG TGGAGATGA-3′), the FAM probe for LMP1 (5′-FAM- GTCATAGTA GCTTAGCTGAAC-3′), EBNA1 forward (5′-TACAGGACCTGGAAATGGCC-3′), EBNA1 reverse (5′-TCTTTGAGGTCCACTGCCG-3′), the FAM probe for EBNA1 (5′-FAM- AGGGAGACACATC TGGACCAGAAGGC-3′), *RPPH1* forward (5′- GTCAG ACTGGGCAGGAGATG-3′), *RPPH1* reverse (5′-TGGC CGTGAGTCTGTTCC-3′), and the HEX probe for *RPPH1* (5′-HEX- TGCCTCCTTTGCCGGAGCTT-3′). The final PCR mixture (10 μl) contained 5 μl of TaqMan universal PCR master mix (Life Technologies), 3 μl of a DNA sample, 1 μl of mixture of *RPPH1* primer, and 1 μl of mixture of LMP1 or EBNA1 primer.

### Statistical analysis

Spearman's correlation coefficients were determined to assess the relationships between the EBV DNA data determined by different methods. Nonparametric tests were performed for subgroup comparisons (Wilcoxon rank-sum test) and for comparisons between paired samples in each subgroup (Wilcoxon signed-rank test). A χ2 test or Fisher's exact test was performed to assess associations between the EBV status and clinicopathological factors. All statistical tests except for the paired tests were two-sided. OS and RFS rates were calculated by the Kaplan–Meier method with the date of gastrectomy as the starting point. Differences in survival rates were examined by the log-rank test. *P* < 0.05 was considered statistically significant.

## SUPPLEMENTARY MATERIALS FIGURES AND TABLES




