# Supplementary material for: Clinical utility of circulating cell-free Epstein–Barr virus DNA in patients with gastric cancer
Source: Oncotarget. 2017 Feb 24;8(17):28796–804. doi: 10.18632/oncotarget.15675 (PMC5438692; doi:10.18632/oncotarget.15675)
Supplement: Supplementary file 1 [file oncotarget-08-28796-s001.pdf]

Clinical utility of circulating cell-free Epstein–Barr virus DNA in patients with gastric cancer

SUPPLEMENTARY FIGURES AND TABLES

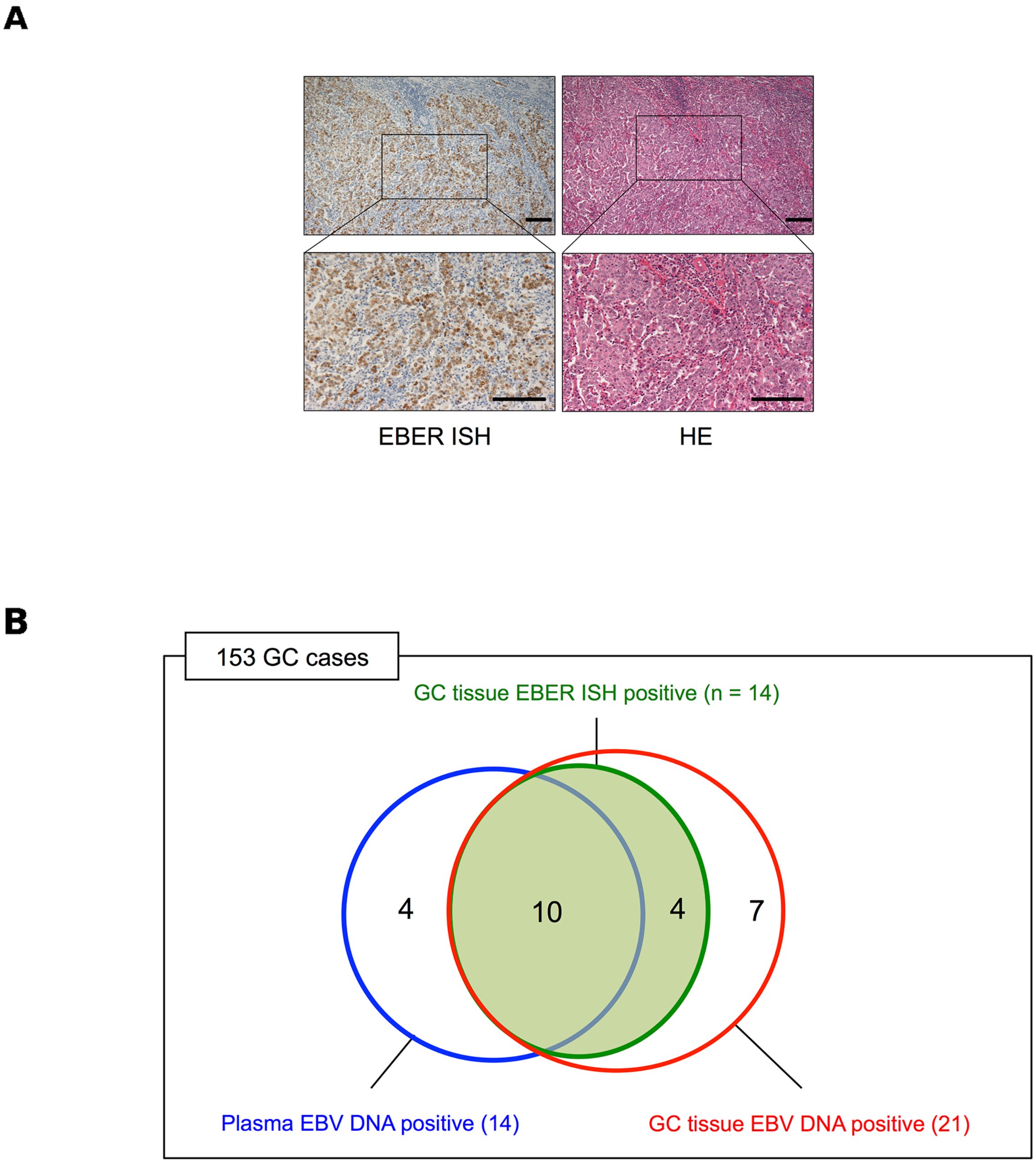

Supplementary Figure 1: Schematic view of data from EBV assays. Representative photomicrographs of an EBV-positive gastric cancer tumor stained with EBER-ISR (left panel and hematoxylin and eosin (right panel). Bar, 40 um b. venn diagram summarizing the overlap among positive cases from plasma EBV rqPCR (left blue circle), tissue EBER (center green circle), and tissue ENV rqPCR (right red circle).

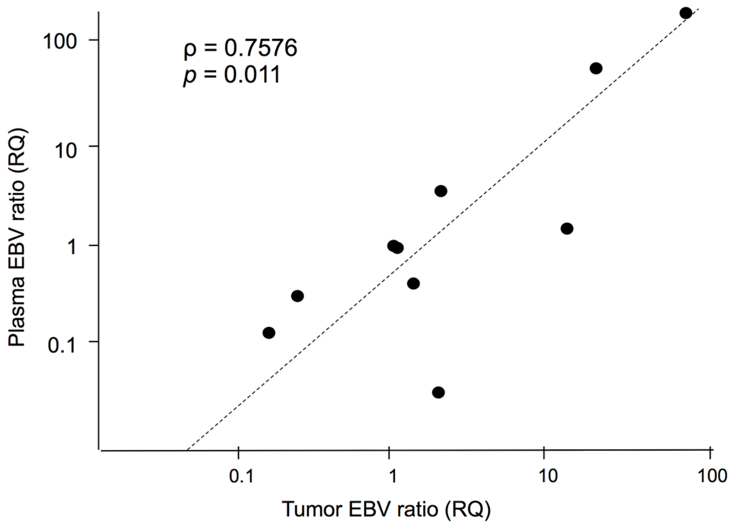

Supplementary Figure 2: Correlation between tissue and plasma EBV ratios in patients with GC. Tissue and plasma EBV rations in GC cases with positive ratios detected in both plasma and tumor sample showed significant correlation (p = 0.7576, p = 0.011, Spearman's analysis).

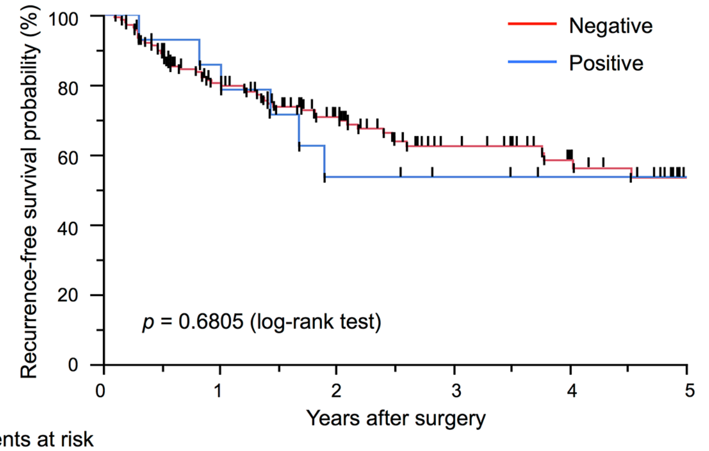

Patients at risk

Negative	139	101	68	40	29	10
Positive	14	13	7	5	3	3

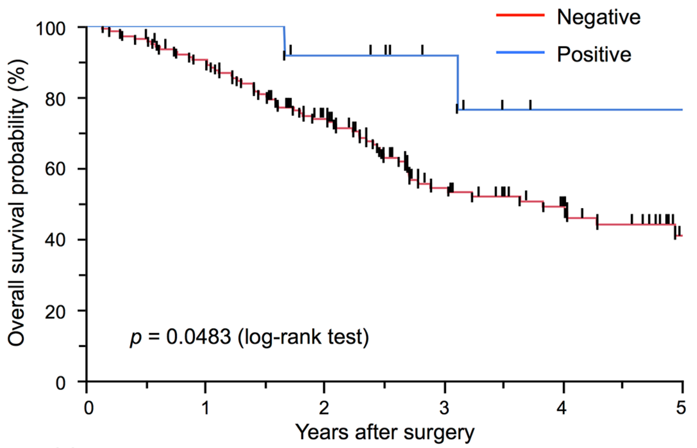

Patients at risk

Negative	139	123	88	47	34	11
Positive	14	14	11	7	3	3

Supplementary Figure 3: Correlation between EBER ISH status and outcome in patients with GC. Kaplan-Meier curves for recurrence-free survival rates (top) and overall survival rates (bottom) of GC patients according to the results of EBER ISR. Log-rank test was used for statistical analysis.

Supplementary Table 1: Plasma LMP1 and EBNA1 DNA in GC with analysis of EBER ISH

Variables	n	Tissue EBER[a]				
		Negative (%)		Positive (%)		*p* value
Total	153	139	(90.8)	14	(9.2)	
Plasma *LMP1* DNA						
negative	138	135	(97.1)	3	(21.4)	**<0.0001**
positive	15	4	(2.9)	11	(78.6)	
Plasma *EBNA* DNA						
negative	139	135	(97.1)	4	(2.9)	**<0.0001**
positive	14	4	(28.6)	10	(71.4)	

p values are from χ^2 or Fisher's exact and the results were considered statisticaly significant at $p < 0.05$.
Statistically significant values are in boldface type.
[a] Tissue EBV was determined by in situ hybridization as described in the text.

Supplementary Table 2: Association between tumor size and plasma EBV DNA in patients with tissue EBV DNA positive and EBER ISH positive

Variables	n	Plasma EBV DNAa		*p* value[d]
		negative	positive	
EBER ISH positive[b]	14	7	7	
Tumor size (mm)				
< 70	7	4	3	0.0699
> 70	7	0	7	
Tissue EBV DNA positive[c]	21	11	10	
Tumor size (mm)				
< 70	11	8	3	0.0861
> 70	10	3	7	

[a] Plasma EBV DNA was determined by real-time quantitative polymerase chain reaction (rqPCR) using latent membrane protein 1 (LMP1) and Epstein–Barr nuclear antigen 1 (EBNA1) primers
[b] Tissue EBV was determined by in situ hybridization as described in the text.
[c] Tumor EBV DNA was determined by rqPCR using LMP1 and EBNA1 primers.
[d] *p* values are from Fisher's exact test.

Supplementary Table 3: Clinicopathological features of 153 gastric cancer (GC) patients with analysis of Epstein–Barr virus (EBV)

See Supplementary File 1
